# Continuous Glucose Monitoring in Transient Neonatal Diabetes Mellitus—2 Case Reports and Literature Review

**DOI:** 10.3390/diagnostics13132271

**Published:** 2023-07-04

**Authors:** Tatiana Chisnoiu, Adriana Luminita Balasa, Larisia Mihai, Ancuta Lupu, Corina Elena Frecus, Irina Ion, Antonio Andrusca, Alexandru Cosmin Pantazi, Maria Nicolae, Vasile Valeriu Lupu, Constantin Ionescu, Cristina Maria Mihai, Simona Claudia Cambrea

**Affiliations:** 1 Department of Pediatrics, Faculty of General Medicine, “Ovidius” University, 900470 Constanta, Romania; 2 Pediatrics, County Clinical Emergency Hospital of Constanta, 900591 Constanta, Romania; 3 Pediatrics, “Grigore T. Popa”, Department of Mother and Child Medicine, University of Medicine and Pharmacy, 700115 Iasi, Romania; 4 Department 1 Preclinical, Faculty of General Medicine, “Ovidius” University, 900470 Constanta, Romania; 5 Department of Infectious Diseases, Faculty of General Medicine, “Ovidius” University, 900470 Constanta, Romania

**Keywords:** neonatal diabetes, glucose monitoring, monogenetic diabetes

## Abstract

Neonatal diabetes mellitus is a rare genetic disease that affects 1 in 90,000 live births. The start of the disease is often before the baby is 6 months old, with rare cases of onset between 6 months and 1 year. It is characterized by low or absent insulin levels in the blood, leading to severe hyperglycemia in the patient, which requires temporary insulin therapy in around 50% of cases or permanent insulin therapy in other cases. Two major processes involved in diabetes mellitus are a deformed pancreas with altered insulin-secreting cell development and/or survival or faulty functioning of the existing pancreatic beta cell. We will discuss the cases of two preterm girls with neonatal diabetes mellitus in this research. In addition to reviewing the literature on the topic, we examined the different mutations, patient care, and clinical outcomes both before and after insulin treatment.

## 1. Introduction

Neonatal diabetes mellitus (NDM), also known as monogenetic diabetes of infancy, is an uncommon condition that most often affects newborns or young children under the age of six months. When a baby at term has intrauterine growth restriction (IUGR) and an early, suboptimal postnatal weight evolution, neonatal diabetes should be considered a differential diagnosis [1].

Currently, there are two categories that are identified based on clinical and genetic factors:

– permanent neonatal diabetes mellitus (PNDM)
– transient neonatal diabetes mellitus (TNDM)

It is estimated that between 1 in 215,000 and 1 in 400,000 newborns have diabetes mellitus [1]. The diabetes in around 50% of these infants is just temporary. According to research, chromosome 6q24 methylation defects (paternal uniparental disomy, paternal duplication, and loss of methylation without a structural defect) account for about 70% of these cases [2]. These conditions are characterized by:

– moderate or severe intrauterine growth restriction
– early development (during the first week of life)
– rare, mild, non-ketotic hyperglycemia

In the southeastern Anatolian area of Turkey, Demirbilek et al. discovered that the total yearly incidence of NDM (including PNDM) is 1 in 30,000 live births (with PNDM 1 in 48,000 live births). In this Turkish cohort, the prevalence of TNDM due to changes in the ZFP57 gene was equally great, with two out of three TNDM patients with anomalies in the methylation of chromosome 6q24 having mutations in the ZFP57 gene. For the reason that there is a 25% chance of recurrence in affected families with such a historical background, TNDM may be suspected. This is due to the occurrence of ZFP57 gene mutations, which correlate with consanguinity [3]. To determine the actual prevalence rate of NDM, further research is required. Given that the pattern of inheritance for the genes involved differs greatly, genetic counseling for NDM patients is dependent on the genetic etiology [4].

Affected men have a 50% probability of transferring TNDM to their descendants in family situations where there is a paternal duplication of the chromosome 6q24 area. Children will not be impacted by maternal duplication, but the boys may be able to pass on TNDM to their children [5]. Few cases of transient diabetes are linked to functional abnormalities of KATP channels on the β-cell membrane (activating mutations of the ABCC8 and KCNJ11 genes), but some clinical characteristics, such as higher birth weight, later onset of hyperglycemia, and relatively delayed remission, may still exist [6]. Recently, other genetic subtypes were discovered, including certain transcription factors involved in pancreatic development (GATA6, PAX6, NEUROG3, and NEUROD1), especially those connected to a phenotypic range that includes extrapancreatic characteristics and diabetes [7].

## 2. Case Presentations

We present two instances of preterm girls who had intrauterine growth restriction (IUGR) (<3rd percentile) and were delivered at 37 weeks and 35 weeks of gestation, respectively. The characteristics of both cases are presented in Table 1.

In the first case, the baby’s blood glucose level increased from 275 mg/dL (on the first day) to 370 mg/dL. For the first few days, the continuous insulin infusion was initiated at a rate of 0.1–0.3 mL/h (0.01–0.03 units/kg/h), and thereafter it was maintained at 0.1 mL/h continuously (0.43 units/24 h).

In the second case, the baby‘s blood glucose levels increased from 350 mg/dL on day one to 467 mg/dL on day two. The insulin infusion was begun continuously at a rhythm of 0.026 units/kg/day and subsequently progressively decreased to 0.006 units/kg/day as glucose levels fell between 50 and 240 mg/dL.

Due to the increased risk of hyperglycemia and glycemic fluctuation, we used continuous glucose monitoring in both cases. The insulin rhythm was regularly changed in response to feedings and blood sugar measurements. We used for CGM The FreeStyle Libre system which is authorized for the control of diabetes in children older than 4 years old. When the FreeStyle Libre system is scanned or “flashed” across the sensor, it displays the interstitial glucose level. Older versions of the FreeStyle system lacked alerts to warn patients of high or low blood sugar. Another method for transmitting glucose to authorized users is to scan the sensor with a mobile phone equipped with near-field communication. The glucose variance over the last 24 h is shown, and trend arrows are shown. The sensor doesn’t require any calibration and runs for 14 days before switching off on its own. It is the least expensive of the current technologies, with accuracy comparable to that of the Dexcom G6. For use in neonatal critical care, the system lacks a license.

**Figure 1:**
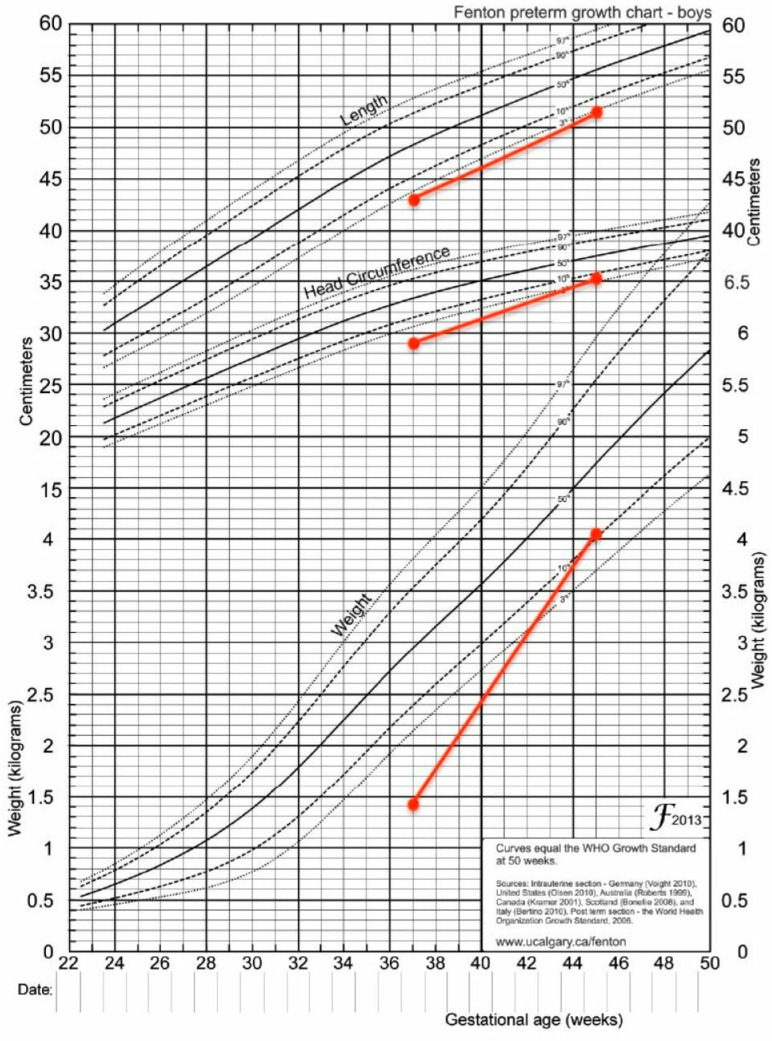
Growth chart-case 1.

**Figure 2:**
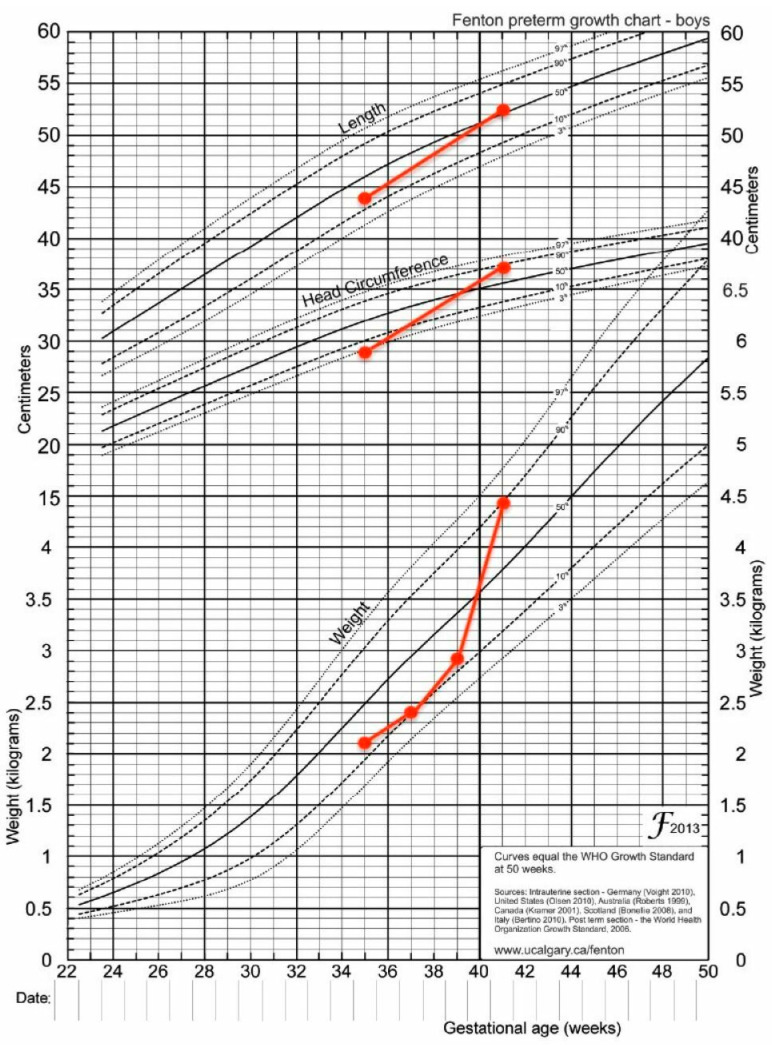
Growth chart-case 2.

In the first case report, after two months, the patient was switched from Insulin drip to continuous and customized doses of rapid-acting Insulin (Lispro). This was completed through an insulin pump whose rhythm was 0.05 units/h for blood glucose levels that varied from 80 to 180 mg/dL. After 2.5 months, the patient was sent home in excellent general health with a weight of 4080 g (Figure 1). With a body weight that exceeded the 50th percentile, the second patient likewise made significant progress (Figure 2).

In both instances, the hyperglycemia was caused by a uniparental disomy at the 6q24 locus (caused by paternal disomy). With the assistance of the University of Exeter, Medical School, Diabetes Genes, we carried out this genetic test. DNA samples from patients with methylation loss were discovered using methylation-specific copy number analysis on the 6q24 gene. A microsatellite study of nine polymorphic chromosome 6 markers revealed no maternal contribution. These findings support the diagnosis of temporary neonatal diabetes caused by paternal uniparental disomy at locus 6q24. 

In both situations, the infants have normal anthropometric profiles, no infectious episodes, and neither hyper- nor hypoglycemia occurrences. The need for long-term follow-up was explained to the parents since there are no clinical, biochemical, or genetic connections to predict a potential recurrence, and the probability of recurrence is not to be ignored.

## 3. Discussion

Neonatal diabetes is a rare condition associated with low birth weight due to dis-turbance of insulin secretion in the intrauterine period [7]. In both of our cases, the birth weight was below the 3rd centile. DNA testing is needed to confirm the molecular etiology of NDM. These tests are expensive and not accessible in Romania, but some centers outside Romania offer them at no cost for research purposes. 

Some patients with neonatal diabetes can be associated with neurological diseases characterized by developmental delay, muscle weakness, seizures, and dysmorphic features [8]. These patients present a type of mutation in the KCNJ11 gene. The catch-up growth is usually present in most cases once the insulin treatment starts, with a good prognosis [9]. In our cases, the growth chart showed that the somatometric evolution was very good. 

Insulin, irrespective of the etiology, is the cornerstone of the therapy of NDM in the early postnatal period. Oral sulfonylurea, which is taken in accordance with the genetic diagnosis of KATP channel mutations, is another viable therapeutic option. The administration of insulin should begin as soon as diabetes is identified by persistent hyperglycemia [10]. We promptly began insulin treatment in both situations.

In practice, fast-acting insulins cost about the same but are more expensive than the previous animal source and biosynthetic regular human insulin preparations. All three can be used in infants, children, adolescents, adults, and the elderly without realizing that they are ideally administered about 15 min before meals to try and control the glycemic fluctuations of most foods and snacks and that they would need to be coupled with some form of basal/background insulin for glycemic control between meals. All fast-acting insulin analogs share the same potential benefits and have minimal differences in terms of clinical performance, reducing nocturnal and postmeal hypoglycemia, and improving coverage for postmeal glycemic excursions [11].

Transient neonatal diabetes mellitus caused by genetic anomalies of the imprinted locus at 6q24 is known as 6q24-related transient neonatal diabetes mellitus, or 6q24-TNDM. Severe intrauterine growth restriction, hyperglycemia (from the neonatal period and before 18 months), dehydration, and the lack of ketoacidosis are the key characteristics [12].

Deafness, severe hypotonia, congenital cardiac problems, renal malformations, and neurologic characteristics including epilepsy may also be present in 6q24-TNDM linked with multilocus imprinting disease (MLID). Diabetes mellitus often starts during the first week of life and lasts three months on average; however, it may last longer, sometimes for up to a year. Although insulin is often required at first, the requirement for it gradually diminishes. Children may have sporadic periods of hyperglycemia, particularly when they are unwell. In adolescence or even dulthood, diabetes mellitus may return. Pregnant women who have experienced 6q24-TNDM are at risk for a recurrence [13].

Docherty et al. investigated the relationship between genotype and phenotype in a global sample of TNDM patients. According to their findings, umbilical hernia (21%) and macroglossia (44%) were the two most prevalent congenital defects. On the other hand, dysmorphic facial features (18%), renal tract anomalies (double kidneys, hydronephrosis, dilated renal pelvis, and vesicoureteral reflux) (9%), cardiac anomalies (ductus arteriosus, tetralogy of Fallot, atrial septal defects, and persistent foramen ovale) (9%), clinodactyly, polydactyly, nail and short finger anomalies (8%), and hypothyroidism (4%) were among the congenital abnormalities that occurred less frequently [14].

Transient neonatal diabetes is an anomaly of the 6q24 locus involving the ZAC (Z finger protein that regulates apoptosis and cell cycle arrest; also known as PLAGL1–pleo-morphic adenoma gene-like 1) and HYAMI genes [15]. The 6q24 variant is also associated with macroglossia and umbilical hernias. The macroglossia was present only in case 2, and the umbilical hernia was present in both our cases. 

A proband with transient neonatal diabetes mellitus and a DNA methylation study revealing relative hypomethylation within the differentially methylated region 6q24 (DMR) is diagnosed with 6q24-TNDM. 

Overexpression of the imprinted genes PLAGL1 and HYMAI on 6q24 leads to 6q24-TNDM. These genes’ shared promoter contains the DMR (i.e., PLAGL1 TSS alt-DMR). Only the paternal alleles of PLAGL1 and HYMAI are expressed under normal circumstances because DMR methylation suppresses the expression of the maternal alleles of these genes [16]. Additional molecular genetic testing may reveal the genetic process at play, which is necessary for genetic counseling. Three distinct genetic mechanisms, including paternal uniparental disomy of chromosome 6 [17], duplication of 6q24 on the paternal allele [18], and hypomethylation of maternal PLAGL1 TSS alt-DMR [19], which results in inappropriate expression of the maternal PLAGL1 and HYMAI alleles, cause duplication of the normal dosage of PLAGL1 and HYMAI (causing 6q24-TNDM).

Shield et al.’s cohort analysis of newborns with TNDM revealed that the median age of initiation of insulin treatment was 12 weeks, the majority of patients were small for gestational age, and the average age of presentation was three days after being born [20]. In TNDM, hyperglycemia is strikingly severe and often accompanied by low or undetectable levels of C-peptide and insulin. Although ketoacidosis may only very rarely result from hyperglycemia, it is more probable in PNDM than in TNDM. More than 95% of TNDM patients have IUGR, which commonly appears in the third trimester [21]. According to 2002 French cohort research, TNDM was linked to higher rates of IUGR (74 vs. 36%) and earlier diagnosis (median age, six days; range, 1–81 days versus median age, 27 days; range, 1–127 days) than PNDM. When compared with individuals with K-ATP channel mutations, patients with TNDM who have abnormalities on chromosome 6q had considerably lower birth weights [22].

It is impossible to utilize low HbA1c (high HbF) as an indication of glycemic control in NDM because of how it is seen in relation to plasma glucose levels [23,24]. The insulin secretion deficit is similar in type 1 and neonatal diabetes. Neonatal diabetes often begins with ketoacidosis. Ketoacidosis from the onset of neo-natal diabetes is treated in the same way as in the case of type 1 diabetes. The child’s life will be saved by insulin treatment, which must be started right away [25].

As neonates are very sensitive to insulin and at risk of developing severe hypoglycemia, insulin treatment should be started with caution. Regular insulin is continuously infused intravenously at 0.05–0.1 U/kg/h and adjusted depending on the blood glucose levels. Therapy aims to restore fluid and electrolyte balance as well as enable tissues to use normal amounts of energy [26].

For newborns with IUGR in particular, insulin therapy is essential to provide optimal weight gain and development; however, treating NDM is challenging since there is a lack of subcutaneous fat, and the need for low doses of insulin. A continuous subcutaneous insulin infusion (CSII), intermittent subcutaneous treatment, or intravenous in-fusion of insulin are several ways in which we can make sure insulin is provided [27]. After the initial treatment of diabetic ketoacidosis, infants with persistent hyperglycemia, despite decreases in glucose infusion rates, and those with persistent glucose excursions should continue receiving regular insulin intravenously, while others are switched to a suitable subcutaneous insulin schedule. Finding a treatment plan that works is often difficult since there is little information on the best insulin doses for young newborns. Patients are switched over to basal insulin injections or a basal/bolus insulin regimen [28].

A significant problem in NDM continues to be the administration of small insulin doses, fluctuating insulin needs, and frequent blood glucose testing. For neonates with diabetes, CSII allows for modest rates of insulin administration [29]. Furthermore, CSII offers more adaptability to account for the variation in oral intake as well as adjustments in energy expenditure as the kid develops. In contrast to injections, CSII is safer, more physiological, simpler to control, and allows for the administration of relatively little insulin [30].

An electrode sensor used in continuous glucose monitoring systems (CGMS) catalyzes the oxidation of glucose, producing an electric current that is measured by a monitor. The interstitial fluid’s glucose level may be continuously measured thanks to the CGMS, which is placed subcutaneously. The sensor is particularly helpful for premature and small gestational-age infants who are susceptible to significant fluctuations in blood glucose levels [31]. When glucose levels are either lower than acceptable limits or higher than acceptable limits, the CGMS is set to provide alarms, allowing the caregiver to react quickly. Since both hypoglycemia and hyperglycemia are linked to immediate neurophysiological abnormalities and long-term neurodevelopmental impairment, timely treatment is crucial from a therapeutic perspective. However, more research is needed as there is not enough experience with its usage or accuracy in infants, despite its claimed benefits for continuous blood glucose testing [32].

According to two recent investigations, needle sensors were also well tolerated in newborns weighing more than 1.5 kg and had no negative breastfeeding effects [33,34]. These trials included newborns as little as 579 g in weight. There were no reports of any severe symptoms, including infection, edema, hemorrhage, or bruising, and the sensors were utilized for up to 7 days with no apparent performance degradation [35,36]. According to Galderisi et al., sensor detachment was a concern in a small number of extremely preterm newborns. However, in the case of two patients, CGM had to be discontinued due to repeated detachments [37].

The evaluation of neonatal hypoglycemia is severely constrained by the fact that glucose concentrations are only provided in the range of 40 mg/dL (2.2 mmol/L) to 400 mg/dL (22 mmol/L), which is a significant restriction of present technology. Point-to-point recalibration of the raw signal, which also enhances the accuracy of CGM in the lower glucose range, may, however, overcome this for retrospective analysis [38]. The sensors’ need for a “wetting” phase, which normally lasts for around 2 h, is another drawback. However, neonates have not been particularly tested to see how long it takes for the signal output to stabilize. According to Harris et al. [39], the mean absolute error peaked on the first day after insertion, which may be partially attributable to increased error during the wetting period.

When converting sensor current to blood glucose concentration, the continuous shift internal algorithm used by CGM is updated by periodic calibration based on blood glucose readings [40]. The three primary types of mistakes that might occur with CGM have some major factors, such as: zero-mean error, drift, and diffusion time delay. Due to the technology used in the sensor and its interstitial location, the zero mean error is the random error of the sensor. The error itself can be rather large. Drift is the term used to describe changes in sensor output between calibration points brought on by biofilm or corrosion on the needle surface, which results in varying currents for the same blood glucose level. While it has not been researched in newborns, the possibility of sensor drift in adults is widely established. A delay is also caused by the diffusion of glucose between the interstitial and vascular compartments. This is around 20 min in lambs [39], which is in line with circumstantial evidence in neonates [34,41,42]. This time delay has the practical effect of making CGM’s positive and negative errors become larger when blood glucose levels increase and decrease, respectively.

The incidence and duration of hypoglycemia in type 1 diabetes have been demonstrated to be decreased using a CGM and insulin pump used together with a computer algorithm (artificial pancreas) [43]. A potential treatment strategy for hyperglycemic preterm newborns is computerized insulin dosage based on predicted insulin sensitivity [43,44]. Although it has been reported that using CGM in conjunction with insulin infusion lowers the incidence of hypoglycemia episodes in a newborn with neonatal diabetes, there is presently no data on the use of CGM to advise insulin therapy in neonatal hyperglycemia [45].

Before CGM may be suggested for real-time monitoring in neonatal intensive care, several obstacles must be resolved. CGM has considerable promise for optimizing blood glucose levels in newborns [40]. In both situations, we successfully employed CGM and saw no negative effects. The last-generation technological innovation, CGM, contributes towards achieving good metabolic control. Up to 67% of all patients with type 1 diabetes used CGM, according to a study conducted in Germany [44]. Reducing hypoglycemia is an important key that can be resolved by assessing the risk factors for problematic hypoglycemia and by introducing advanced diabetes technologies into the management of diabetes [45].

Once NDM is detected, glycemic management and the avoidance of both hyper- and hypoglycemia are the main therapy objectives. Real-time CGM can be a helpful tool for preserving secure glucose control during insulin treatment [46]. 

Prolonged or severe hyperglycemia or hypoglycemia can be lessened using CGM. The best glucose goals, how to achieve them, and the possible impact on long-term health outcomes will need to be determined by more research utilizing CGM [47].

The future of managing diabetes is undoubtedly CGM. Any young children, including neonates, babies, and preschoolers, as well as any kids of any age with cognitive or neurodevelopmental issues that affect their capacity to detect or react to hypoglycemia, should be given CGM consideration [48]. 

The reduction of hypoglycemia, in particular the minimization of severe hypoglycemia, is a goal in the treatment of children and adolescents with type 1 diabetes. Assessing the risk factors for developing severe hypoglycemia is of great importance in preventing dangerous hypoglycemia from occurring [49]. The National Institute for Clinical Excellence (NICE) now recommends that adults and children with diabetes who are at risk of hypoglycemia should use CGM. Hypoglycemia is common in the neonatal period and a preventable cause of poor neurodevelopmental outcomes. Newborn studies have shown that CGM can detect clinically silent hypoglycemia, which has been associated with reduced executive and visual function in early childhood [49].

All neonates with NDM should be fed a high-calorie diet and given enough insulin to support healthy weight gain and development. The amount of carbohydrates in normal human milk and regular baby formula is comparable (about 70 to 75 g/L), but the amount of carbohydrates in enriched human milk is very small (0.1 g/packet). To address calorie requirements and carbohydrate counts, dietary management includes a team of professionals that can advise on this matter [6]. We provide all the information available to encourage exclusive breastfeeding and to develop programs that promote a balanced diet for infants throughout the first year of life [50]. Therefore, carbohydrate estimation for breastfed infants can be challenging. If a patient is fed pumped breast milk, the carbohydrate content can be estimated at 2.1 g per ounce of breast milk. Resources are available to help caregivers estimate the amount of breast milk and, subsequently, the carbohydrates consumed [51].

Around 50% of neonatal diabetes cases see a spontaneous remission, often within the first three months (transient diabetes). However, a clinical return is always a possibility. For the remaining 50%, permanent neonatal diabetes requires a lifetime of medication to regulate glycemia [51].

After puberty, TNDM sufferers frequently experience relapses. Relapsed 6q24-related diabetes is often seen in non-obese, autoantibody-negative patients and is no longer a transitory condition [49]. A considerable portion of patients with relapsing diabetes mellitus (DM) after adolescence have been reported to have insulin insufficiency but not obesity [50]. The first instance of relapsing 6q24-TNDM with predominant insulin resistance is reported by Uchida et al. [52]. 

Many 6q24-TNDM individuals experience recurrent DM after puberty and are born small for gestational age (SGA) [53]. SGA and puberty are well-known risk factors for increasing insulin resistance. According to Docherty LE et al., individuals with 6q24-TNDM had mean (SD) birth weights and gestational weeks of 2001 (417) g and 37.8 (2.7) weeks, respectively [14].

Metz et al. describe in a large cohort of patients with TNDM and PNDM, that five TNDM patients experienced the onset of permanent insulin-dependent diabetes after the age of 8, highlighting the importance of continuing follow-up. The 19 TNDM patients who underwent testing had two paternal isodisomies of chromosome 6, seven paternally-derived trisomies from four families, and two methylation defects in the 6q24 area [22].

Transient neonatal diabetes subtypes 6q24/TNDM and KATP/TNDM have unique clinical characteristics that are caused by various disease processes [54].

In a recent study completed in 2016, Besser et al. included 750 children who had diabetes that had started before they were six months old. The authors said that out of this cohort, 604 patients were born at or before 37 weeks’ gestation, whereas the other patients (n = 146) were [55,56]. 

A number of papers have noted people with 6q24-related diabetes who do not have a history of TNDM [57]. Table 2 summarizes the gene mutations involved in transient neonatal diabetes mellitus.

Expanded methods of genetic assessment for TNDM have been explored as molecular testing develops. A new study investigated 1020 neonatal diabetes patients utilizing a multigene strategy that comprised sequencing of every known gene linked to neonatal diabetes as well as 6q24 methylation tests. Their findings show that this testing technique successfully revealed a genetic reason in 80% of individuals, enabling a more precise prediction of the clinical course, anticipation of subsequent consequences, and the application of targeted medication [59,60].

We’ve come a long way in less than 200 years from not being able to measure blood glucose to being able to provide individuals with diabetes with continuous blood glucose monitoring coupled with continuous subcutaneous insulin infusion [61].

Most newborns with newly diagnosed diabetes require immediate insulin treatment to treat or avoid acute metabolic decompensation and to enable weight growth [62]. The cost-effectiveness of genetic testing for neonatal diabetes responsive to sulfonylurea (SU) has also been examined, in addition to the advantages of sulfonylurea therapy over insulin therapy that have already been covered [63]. 

The variance in individual and SU doses causes variations in the phenotypic profile and responsiveness to therapy for the same mutant genotype. Additionally, the prognosis of neurodevelopmental abnormalities in NDM with foci of encephalomalacia may be improved by SU only to a limited extent [64]. Our understanding of human illnesses has been considerably enhanced by developments in genomic medicine. Phenome, however, is not fully comprehended. The processes behind newborn disorders have been better understood thanks to high-resolution and multidimensional phenotypes, which may also help to improve treatment approaches [65].

The low prevalence of TNDM (1:200,000 to 1:400,000 live births) makes it difficult to gather information regarding its clinical characteristics, prognosis, and treatment. Until now, modest case studies have been used to characterize the clinical characteristics of 6q24 TNDM, some of which included patients without a molecularly verified diagnosis [14]. 

The majority of individuals with 6q24-TNDM had small-for-gestational-age (SGA) birth weights of 1930 g at 39 weeks of gestation or −2.5 SDS (standard deviation score), which most likely indicates an insulin deficit in utero [50]. 

The patents that have been filed for the use of natural substances to control diabetes lack comprehensive research [66].

## 4. Conclusions

Clinically, therapeutically, and genetically, neonatal diabetes continues to be a difficult condition to treat. There are no recommendations for managing diabetes during the first few months of birth; however, these two points are indisputable recommendations:

○ The availability of genetic results (in a few weeks) significantly altered the short-and long-term management of these infants.
○ These babies need insulin with adequate or high caloric intake to ensure satisfactory weight gain. Insulin administration can now partially mimic the pancreas physiology.

A molecular genetic diagnosis is advised for all NDM patients. These newborns with moderate or severe growth restrictions need early identification and medical therapy (constant insulin infusion and high caloric intake) to prevent catastrophic metabolic issues and allow for appropriate weight gain and brain development. 

Regular follow-up is strongly advised, especially in the early years of childhood, since common illnesses may produce symptomatic hypoglycemia or recurrent hyperglycemia. CGM brought a benefit in this case because we were able to avoid the events of hypoglycemia and act by decreasing the rate of insulin administration.

## Figures and Tables

**Table 1:** Clinical characteristics and laboratory results of the patients.

Patient	Case Report 1	Case Report 2
Gender	F	F
Age	First day of life	First day of life
Birth weight	1480 g—37 weeks of gestation Severe Intrauterine growth restriction IUGR < 3rd percentile	2100 g—35 weeks of gestation Intrauterine growth restriction (Oligohydramnios)
Length	43 cm	44 cm
Head circumference	29 cm	31 cm
Feeding type	Formula-fed up to 13 days of life, then breastfed	Mixed fed—breastfed and formula fed since the first day of life
Clinical features	facial dysmorphism epicanthus umbilical hernia	facial dysmorphism epicanthus macroglossia pectus excavatum umbilical hernia short hallux
Glucose level at diagnosis	275 mg/dL	350 mg/dL
Genetic test	Uniparental disomy at the 6q24 locus	Uniparental disomy at the 6q24 locus
Treatment	Insulin Lispro was started continuous with a rhythm between 0.1–0.3 mL/h (0.01–0.03 units/kg/h) for the first days and constantly 0.1 mL/h after that (0.43 units/24 h); At 2 months—insulin pump with a dose 0.05 units/h	Insulin Lispro was started continuous with a rhythm between 0.026 units/kg/day and then reduced gradually up to 0.006 units/kg/day;
Evolution	Discharge after 2 months and 2 weeks Lispro Insulin dose was reduced gradually and subsequently was stopped one week following her discharge; At the age of 12 months, the baby develops very well, normal anthropometric parameters, no infectious episodes and no hyper- or hypoglycemic events.	Discharge at 2 months when treatment with Lispro insulin was stopped; At age of 6 months, the baby develops very well, with normal anthropometric parameters, no infectious episodes and no hyper- or hypoglycemic events.

**Table 2:** Gene mutations in transient neonatal diabetes mellitus and Therapy [58].

Mechanism of β-Cell Dysfunction	Gene Mutation	Chromosome Locus	Inheritance	Additional Features	Therapy
Reduced β-cell development	*ZAC* (*IPLAG1*)/*HYMA1*	6q24	imprinting; AD	macroglossia; umbilical hernia	insulin
	*ZEP57*	6p22.1	AR		insulin
	*HNF1B*	17q21.3	AD	pancreatic cysts hypoplasia; renal	
Failure membrane to depolarize	*KCNJ11* (Kir6.2)	11p15.1	AD; de novo	low developmental birth weight; delay; DEND	sulfonylurea
Failure channel to close KATP	*ABCC8* (*SUR1*)	11p15.1	AD; AR; de novo	low birth weight	sulfonylurea
Abnormal β-cell function	*INS* (proinsulin)	11p15.5	AR	low birth weight	Insulin

## Data Availability

The data is available on request from the corresponding author.
